# Magnesium Ion-Mediated Regulation of Osteogenesis and Osteoclastogenesis in 2D Culture and 3D Collagen/Nano-Hydroxyapatite Scaffolds for Enhanced Bone Repair

**DOI:** 10.3390/jfb16100363

**Published:** 2025-09-29

**Authors:** Sílvia Sá Paiva, Avelino Ferreira, Eavan Pakenham, Kulwinder Kaur, Brenton Cavanagh, Fergal J. O’Brien, Ciara M. Murphy

**Affiliations:** 1 Tissue Engineering Research Group (TERG), Department of Anatomy & Regenerative Medicine, RCSI University of Medicine and Health Sciences, D02 YN77 Dublin, Ireland; silviapaiva@rcsi.ie (S.S.P.);; 2 Advanced Materials and Bioengineering Research Centre (AMBER), Trinity College Dublin (TCD), D02 PN40 Dublin, Ireland; 3 School of Pharmacy & Biomolecular Sciences, RCSI University of Medicine and Health Sciences, D02 YN77 Dublin, Ireland; 4 Cellular and Molecular Imaging Core, RCSI University of Medicine and Health Sciences, D02 YN77 Dublin, Ireland; 5 Trinity Centre for Biomedical Engineering, Trinity College Dublin (TCD), D02 PN40 Dublin, Ireland

**Keywords:** magnesium, collagen–nanohydroxyapatite scaffolds, bone regeneration, osteogenesis, osteoclastogenesis

## Abstract

Bone regeneration depends on a delicate balance between osteoblast-driven bone formation and osteoclast-mediated resorption, coordinated by complex biochemical cues. Magnesium (Mg^2+^) is known to modulate these processes. However, despite extensive research, its ability to simultaneously enhance osteogenesis and inhibit osteoclast activity remains unclear. In this study, we first investigated the effect of extracellular Mg^2+^ (0, 5, 10, 25, 50 mM) on osteoblast and osteoclast differentiation in 2D culture to determine whether a single Mg^2+^ dosing regimen can simultaneously promote osteogenesis while inhibiting osteoclast differentiation and maturation. A concentration dependent effect of Mg^2+^ was observed on both cell types, with increasing Mg^2+^ concentrations up to 25 mM significantly reducing osteoclast formation yet concurrently inhibiting osteogenic differentiation. At 50 mM, Mg^2+^ exhibited cytotoxic effects on both cell types. We then leveraged the osteogenic properties of biomimetic collagen/nano-hydroxyapatite (Coll/nHA) scaffolds by incorporating Mg^2+^ into the nHA phase to enable localised, controlled delivery. At a scaffold-loaded equivalent of 25 mM Mg^2+^, we observed enhanced bone matrix deposition alongside reduced osteoclast maturation, indicating a synergistic effect between Mg^2+^ and nHA in promoting osteogenesis. While no optimal synergistic dose was identified in 2D culture, these findings demonstrate that Coll-nHA scaffolds offer a promising strategy for localised Mg^2+^ delivery to enhance osteogenesis and suppress osteoclastogenesis. Importantly, the ease of scaffold modification opens the door to incorporating additional bioactive molecules, further advancing their potential in bone tissue engineering applications and the development of next-generation biomaterials for bone regeneration.

## 1. Introduction

Bone is constantly renewed and repaired through a tightly regulated balance between osteoclast-mediated bone resorption and osteoblast-mediated bone formation [1]. Several factors, including systemic hormones, protein growth factors, ions, and trace elements, have been shown to play a vital role in regulating this balance, influencing both the recruitment and differentiation of osteoclasts and osteoblasts [2,3]. The presence of these factors at bone defect sites enables the activation of specific signalling pathways, enhancing the expression of essential genes to control the rate of bone resorption and formation, ensuring effective bone repair [2,4,5]. Previous studies indicated that the local accumulation of bioactive molecules capable of influencing bone cell behaviour provides crucial extracellular signals to which osteoclasts and osteoblasts respond in a time- and concentration-dependent manner [4,6,7,8,9]. Therefore, understanding the spatiotemporal therapeutic effect of such bioactive cues is crucial in achieving a suitable molecular milieu for enhanced bone regeneration.

A number of bioinorganic metallic ions, including silicon (Si), zinc (Zn), magnesium (Mg), copper (Cu), and strontium (Sr), are trace elements of bone, essential to bone metabolism, and play vital roles in up-/downregulating cellular functions and biological processes involved in bone remodelling [10]. Magnesium (Mg^2+^), in particular, is an attractive therapeutic ion that improves the osteoinductive and bioactive properties of biomaterials due to its participation in many biological processes [11]. In vivo, Mg-based implants rapidly degrade and generate a locally concentrated Mg^2+^ microenvironment that favours a potent osteogenic milieu that induces bone formation and growth, and accelerates bone regeneration [12,13]. Moreover, evidence of a reduced osteoclast number at the peri-implant regions point to the ability of Mg^2+^ to decrease osteoclast maturation and activity [14]. However, the optimal Mg^2+^ concentration to simultaneously successfully drive bone formation and reduce bone resorption remains poorly defined.

Biomimetic scaffolds are attractive systems for tissue engineering applications, as they can be finely tuned in order to closely mimic native ECM architecture. In addition, they can also be used as effective delivery systems, as the release rate can easily be modulated based on the scaffold properties (porosity, pore size, crosslinking, etc.) [15]. Type I collagen–hydroxyapatite composite scaffolds are particularly attractive as bone substitutes, not just because they are composed of the two main components of native bone, but also due to their ability in providing an appropriate ECM-like environment that supports cell attachment, proliferation, differentiation, and new bone tissue formation. Hydroxyapatite (HA) is the most extensively studied bioceramic for bone regeneration, due to its chemical and structural similarity to biological mineral apatite [16,17,18]. Although not very soluble, the HA surface provides nucleating sites for precipitation of apatite crystals in culture medium and in body fluids, which contributes to its known bioactivity [19,20]. Biological apatite deviates from stoichiometric HA due to its numerous ionic substitutions on either the Ca cationic positions (Mg^2+^, Zn^2+^, Sr^2+^), the PO_4_^3−^ anionic sites (HPO_4_^3−^, CO_3_^2−^, SiO_4_^3−^), or the OH^−^ anionic sites (CO_3_^2−^, F^−^) together with the presence of ion vacancies in the crystal lattice. This makes HA an attractive material for further functionalization. In particular, nanoscale HA (nHA) has recently been the focus of bone engineering research, as it better mimics the structure of bone mineral, thus improving the biological and resorption behaviour compared to microscale HA. The nanostructure allows a higher surface-to-volume ratio, favouring adhesion, proliferation, and differentiation of osteoprogenitor cells, thereby enhancing osteointegration and deposition of apatite mineral on its surface better than microcrystalline HA, leading to enhanced formation of new bone tissue within a short period. Moreover, nHA is easier to produce, as it requires lower sintering temperatures, making it a material with high potential for bone biomimetic scaffold development [21,22,23,24].

This study focuses on two main aims. Firstly, we sought to elucidate the effect of a concentration range (0, 5, 10, 25, 50 mM) of extracellular Mg^2+^ on osteoblast and osteoclast differentiation over time in 2D culture, to determine whether a single concentration could simultaneously enhance osteogenesis while suppressing osteoclast activity. Second, we explored the use of collagen–nHA scaffolds as a controlled Mg^2+^ delivery platform, investigating their potential to synergistically promote bone regeneration by modulating cell behaviour in a biomimetic 3D environment. This dual approach, combining detailed dosage characterisation with scaffold-mediated ion delivery, provides new insights into harnessing magnesium’s multifunctional role for advanced bone repair strategies.

## 2. Materials and Methods

### 2.1. Expansion of Pre-Osteoblast MC3T3-E1 and Osteoclast Progenitors RAW 264.7 Cells

The murine calvarial pre-osteoblast MC3T3-E1 Subclone 4 (CRL-2593^TM^, ATCC, Manassas, VA, USA) cell line was chosen due to it providing a lineage-committed cell population devoid of phenotypic and functional differences to investigate the influence of Mg^2+^ on osteogenic differentiation [25,26]. MC3T3-E1 were expanded in growth medium consisting of α-MEM supplemented with 10% foetal bovine serum, 1% L-glutamine, and 1% Pen/Strep. Cells were maintained in culture under standard growth conditions for 5 to 7 days at 37 °C, 5% CO_2_, and 90% humidity, with fresh media changes every 2–3 days. At ~80% confluency, cells were detached with 0.25% trypsin-EDTA and centrifuged at 1200 rpm for 5 min at room temperature. The cells were then passaged at a subcultivation ratio of 1:3 to 1:6.

Adult murine male monocyte/macrophage cell line RAW 264.7 (TIB-71^TM^, ATCC, USA) was selected as a precursor model for the generation of osteoclast-like cells in vitro [27,28]. RAW 264.7 cells were expanded in growth medium consisting of α-MEM supplemented with 10% heat-inactivated FBS, 1% L-glutamine, and 1% Pen/Strep. Cells were maintained in culture under growth medium conditions for 5 to 7 days at 37 °C, 5% CO_2_, and 90% humidity, with fresh media changes every 2–3 days. At ~60% confluence, cells were detached with a cell scraper, centrifuged at 1200 rpm for 5 min at room temperature, and passaged at a subcultivation ratio of 1:3 to 1:6.

### 2.2. Optimisation of Mg^2+^ Concentrations for MC3T3-E1 and RAW 264.7 Cells Culture

Mg^2+^ concentrations 0.5, 1, 10, 25, and 50 mM were selected based on previous studies to determine an optimal dosage range [29,30,31,32,33]. MgCl_2_ was used as Mg^2+^ precursor due to its high solubility in culture medium. Mg^2+^ concentrations were added to growth medium and sterile filtered using a 0.2 µm sterile PVDF filter (Fisher Scientific, Waltham, MA, USA).

MC3T3-E1 and RAW 264.7 cells were seeded in 12-well plates at a density of 3000 cells/cm^2^ in the respective growth medium. After 24 h, the media was removed and replaced with growth media supplemented with increasing concentrations of Mg^2+^. Cells were maintained under Mg^2+^ exposure for 7 days, with fresh Mg^2+^ stimulation every 2–3 days. Cell proliferation and metabolic activity were assessed 1, 3, and 7 days after exposure.

#### Cell Proliferation and Metabolic Activity

The proliferation of pre-osteoclasts and osteoblasts exposed to various Mg^2+^ concentrations was assessed using Quant-iT^TM^ PicoGreen^TM^ dsDNA Assay kit (Invitrogen^TM^, Carlsbad, CA, USA). At every desired time point, cells were washed twice with PBS, lysed with 500 μL of 1X assay buffer in dH_2_O containing 1% (*v*/*v*) Triton X-100, and stored at −20 °C. Three freeze–thaw cycles were performed to ensure the complete break of cell membrane and release of DNA. Samples were diluted 1:25 in assay buffer and assayed in duplicate according to the manufacturer’s instructions. A DNA-free well was included as a blank. Fluorescence was read at excitation/emission wavelengths of 480/520 nm in a microplate reader (Tecan Infinite^®^ 200 Pro, Morgan Hill, CA, USA), equipped with i-control^TM^ software (v2.0). Values of average fluorescence of blank samples were subtracted from fluorescence values of experimental samples, and DNA concentrations weredetermined using a standard curve. Results are presented as DNA content (ng/mL). AlamarBlue^TM^ cell viability reagent (DAL1100, Invitrogen^TM^) was used to assess cell metabolic activity. At every desired time point, media was removed and replaced with 500 μL of fresh complete growth media containing 10% AlamarBlue^TM^. Cells were incubated for 2 h, protected from direct light, at 37 °C. A cell-free well was included as a blank. A total of 100 μL of the supernatant was transferred to a black 96-well plate in triplicates, and fluorescence was measured at an excitation/emission wavelength of 540/580 nm. Blank values were subtracted from experimental values, and results were presented as the percentage in metabolic activity normalised to the control group (Media Only) at day 1. A reduction in cell metabolic activity of over 25% was considered a cytotoxic effect, in accordance with ISO 10993-5 [34].

### 2.3. Effects of Magnesium on Osteogenesis

MC3T3-E1 cells were seeded in 6- or 12-well cell culture plates at a density of ~3000 cells/cm^2^ in complete growth medium. After 24 h, the media were removed and replaced with either growth or osteogenic media, supplemented with different Mg^2+^ concentrations. Cells were maintained under Mg^2+^ exposure for 28 days, with fresh Mg^2+^ stimulation every 2–3 days. Media Only and Osteogenic Media without Mg^2+^ supplementation were used as negative and positive controls for osteogenic differentiation, respectively. All experiments used cells within passages 12 to 16.

#### 2.3.1. ALP Activity and Staining

The differentiation activity of MC3T3-E1 was analysed by measurement of ALP activity and histochemical staining at 3-, 7-, and 14-days post exposure. To assess ALP activity, cells were lysed, as described above, for DNA quantification and ALP activity was measured using Sensolyte^®^ pNPP Alkaline Phosphatase Assay kit (AnaSpec, Cambridge Bioscience, Cambridge, UK). The cell lysates were diluted 1:2 in an assay buffer and assayed in duplicate, according to the manufacturer’s instructions. The reaction occurred for 30 min before absorbance was recorded for endpoint reading at 405 nm. Blank values were subtracted from experimental values, and ALP activity was determined using a standard curve.

To stain for ALP activity, cells were fixed in 10% formalin for 15 min and washed thrice in PBS for 3 min. Cells were incubated with 500 μL of ALP buffer containing 100 mM Tris, 50 mM MgCl_2_ and 100 mM NaCl in dH_2_O at room temperature, in an opaque light box, for 10 min. ALP buffer was then removed, and 500 μL of ALP substrate containing 1X Fast Red and 1X Naphthol AS-MX phosphate in ALP buffer was added to cells for ~15 min. ALP substrate was removed, and a series of washes were repeated before blot drying. Coverslips were mounted with a solution of 50% glycerol/Hoechst, and cells were visualised under a fluorescence microscope.

#### 2.3.2. Assessment of Mg^2+^ Matrix Mineralisation

Calcium deposition was assessed by Alizarin Red staining 28 days post exposure. Cells were fixed, as described previously, and incubated with 800 μL of 2% Alizarin Red S staining solution (32366, ScienceCell, Carlsbad, CA, USA) for 25 min at room temperature. Several washes with dH_2_O were performed to remove excess of dye and areas of mineralised matrix were visualised under a light microscope.

#### 2.3.3. Gene Expression of Osteoblast Markers

Gene expression analysis of osteoblast markers was performed using the real-time quantitative Reverse Transcription-Polymerase Chain Reaction (qRT-PCR). At 3-, 7-, and 14-days post exposure, cells were washed twice in PBS and lysed with 500 μL QIAzol lysis reagent (Qiagen, Venlo, The Netherlands). Total RNA was extracted using the RNeasy Mini kit (74106, Qiagen), according to manufacturer’s instructions. A single silica membrane was loaded with a poll of three wells from each individual test sample. Extracted RNA was eluted in molecular grade H_2_O. Quantification and quality analysis of purified RNA was performed using NanoQuant Plate^TM^ (Tecan, USA), with absorbance recorded at 260/280 nm. For each test sample, 800 ng of purified RNA were reverse transcribed to complementary DNA (cDNA), using a QuantiTect Reverse Transcription kit (205314, Qiagen) and a MiniAmp^TM^ Thermal Cycler (Applied Biosystems, ThermoFisher, Waltham, MA, USA), and following manufacturer’s instructions. cDNA samples were diluted to a final concentration of 5 ng/μL with RNase-free dH_2_O and added to PCR plates in duplicate. Samples were mixed with SensiMix SYBR Green Low-ROX kit (Medical Supply Co., Dublin, Ireland) and with primer sets (Qiagen) specific to each target. qRT-PCR reactions were performed in a QuantStudio^TM^ 5 Real-Time PCR System (Applied Biosystems, ThermoFisher, USA) as follows: 95 °C for 3 min, followed by 40 cycles at 95 °C for 1 min, 56 °C for 1 min, and 72 °C for 30 s, and a final extension time at 72 °C for 10 min. The mRNA expression levels for each gene of interest were calculated using the ΔΔCt method relative to the reference housekeeping gene GAPDH or GAPDH/18S average. Results were normalised to Media Only at day 3 and are presented as mRNA fold change in expression. Genes with fold change values >1 were considered upregulated.

### 2.4. Assessment of Mg^2+^ on Osteoclast Differentiation

RAW 264.7 cells were seeded in 12- or 6-well cell culture plates at a density of ~3000 cells/cm^2^ in complete growth medium. After 24 h, the media were removed and replaced with RANKL-induced media supplemented with Mg^2+^. Cells were maintained under Mg^2+^ exposure for 7 days, with fresh Mg^2+^ stimulation every 2–3 days. Growth medium and RANKL-induced medium without Mg^2+^ supplementation were used as a negative and a positive control for osteoclast differentiation, respectively.

#### 2.4.1. Effects of Mg^2+^ Concentration on Osteoclast Markers Expression

Gene expression of osteoclast markers was analysed at days 3 and 5 post exposure using qRT-PCR, as described previously, with the exception of purified RNA mass—500 ng rather than 800 ng were used.

#### 2.4.2. Effect of Mg^2+^ in RANKL-Derived Osteoclast Fusion

The formation of multinucleated osteoclast-like cells of RANKL-induced RAW 264.7 with or without Mg^2+^ stimulation was monitored under anOlympus IX51-AnalysisIS light microscope (Olympus Scientific Solutions Americas, Essex, UK) over 5 days of culture. The number of multinucleated cells (MNCs) (≤2 nuclei) and osteoclasts (≥3 nuclei) per field of view per condition were manually counted using ImageJ (v2.16.0).

#### 2.4.3. Tartrate Resistant Acid Phosphatase (TRAP) Staining and Activity

The differentiation activity of RANKL-induce RAW 264.7 was analysed by measurement of TRAP activity and histochemical staining at days 3 and 5 post exposure. Cells were fixed, as previously described, and stained for TRAP using Leukocyte Acid Phosphatase kit (387-A, Sigma-Aldrich, Burlington, MA, USA) for 45 min at 37 °C, following manufacturer’s instructions. Purple-stained TRAP-positive (TRAP^+^) cells were visualised under a light microscope and the number of TRAP^+^ MNCs per field of view per condition was determined using ImageJ. TRAP enzymatic activity was measured in cell culture supernatants. TRAP substrate solution containing 10 mM *p*NPP in substrate buffer (40 mM sodium tartrate, 50 mM acetic acid, pH 4.8) was prepared at time of assay. At days 3 and 5 of culture, 50 µL of each individual sample were mixed with 50 µL of TRAP substrate solution and incubated for 45 min, protected from light, at 37 °C. The reaction was stopped and *p*NPP converted to *p*-nitrophenol by adding 50 µL of 0.2 M NaOH. Absorbance was read at 405 nm, and results were normalised to absorbance values of known concentrations of *p*-nitrophenol. One unit of TRAP activity corresponds to 1 µmol *p*-nitrophenol liberated per minute of reaction. Results are expressed as a concentration of TRAP activity (µmol/mL).

#### 2.4.4. F-Actin Fluorescence Staining

After 5 days of exposure, cells were fixed and blocked with 2% FBS in PBS for 20 min, and stained for cytoskeleton with 1:600 diluted Alexa Fluor^TM^ 546 phalloidin (A22283, ThermoFisher) overnight at 4 °C. The dye was then removed, cells were washed 3x with PBS for 5 min, and nuclei were counterstained with 1 mg/mL of Hoechst for 5 min. Cell morphology and actin ring formation were visualised under a fluorescence microscope. The number of actin rings, osteoclasts (OC), and nuclei of the largest OC, as well as the area of the largest OC per field of view per condition, were quantified using ImageJ.

### 2.5. Scaffold Fabrication and Characterisation

#### 2.5.1. nHA-Mg Particles Synthesis

nHA and Mg^2+^-substituted nHA particles were prepared following a wet chemical precipitation reaction adapted from the literature [35]. Ca(NO_3_)_2_·4H_2_O, (NH_4_)_2_HPO_4_, and MgCl_2_ were utilised as Ca, P, and Mg precursors, respectively. All reactions were performed at room temperature. Briefly, a 100 mL solution of 1 M Ca(NO_3_)_2_·4H_2_O and 100 mL solution of 0.6 M (NH_4_)_2_HPO_4_ were prepared in freshly distilled water and stirred for 1 h. Then, the (NH_4_)_2_HPO_4_ solution containing 200 µL of dispersing agent Darvan was slowly added to the Ca(NO_3_)_2_·4H_2_O solution [36]. The pH of the solution was adjusted to 9.8 with 2 M NaOH in order to control particle size within the nanorange. For Mg^2+^-substituted nHA particles, the Mg^2+^ precursor was added into the Ca(NO_3_)_2_·4H_2_O solution, according to the desired stoichiometric ratios (see Table 1 below). A stoichiometric (Mg + Ca)/P ratio of 1.67 was maintained throughout all reactions.

The resulting suspensions were left under continuous stirring overnight and then centrifuged for 15 min at 12,000× *g* to produce a solid white precipitate. Precipitates were washed several times in dH_2_O to remove residual NH_4_NO_3_ and HNO_3_ from the synthesis of nHA and Mg/nHA, respectively. The obtained precipitates were freeze-dried in a FreeZone benchtop freeze-dryer (Labconco, Michigan, MI, USA) for 36 h to remove excess water. The aggregated powder was further heat dried in a vacuum oven (VacuCell, MMM, Planegg, Germany) for 24 h at 150 °C. Final powders were sieved through a 120 µm mesh.

#### 2.5.2. Scaffolds Fabrication

Collagen, collagen-nHA (Coll/nHA), and Mg^2+^-functionalised Coll/nHA (Coll/nHA-10 mM and Coll/nHA-25 mM) composite scaffolds were fabricated using a freeze-drying protocol previously developed in our lab [37,38]. Briefly, collagen slurries (0.5% *w*/*v*) were made by adding type I bovine collagen to a 0.05 M glacial acetic acid solution, blended at 15,000 rpm using an Ultra Turrax T18 overhead blender (IKA Works Inc., Wilmington, NC, USA), and maintained at 4 °C until a homogenous slurry with complete collagen dissolution was obtained. For bioceramic-incorporating collagen scaffolds, synthesised nHAp and Mg/nHAp particles were resuspended in 3 mL of a 0.1 M acetic acid solution and slowly added to collagen slurry (50% *w*/*w*) under continuous blending. The pH of the solution was maintained at 4.5. After blending, slurries were degassed using a vacuum pump and transferred into a custom-built stainless-steel mould (400 µL/well, internal dimension of discs: 9.5 mm ⌀ × 5 mm height) and lyophilised in an Epsilon 2–4 LSCplus freeze dryer (Martin Christ, Harz, Germany) at a constant cooling rate of 1 °C/min to a final temperature of −40 °C. Three batches of scaffolds were prepared.

Freeze-dried scaffolds were dehydrothermally (DHT) cross-linked in a vacuum oven for 24 h at 105 °C and 0.05 bar [39,40]. Scaffolds were then chemically cross-linked by the glutaraldehyde (GTA) vapour crosslinking method [41]. Briefly, after DHT, scaffolds were disposed into histological cassettes and exposed to a solution of 25% GTA for 6 h in a vacuum desiccator. At the end of reaction, scaffolds were air dried overnight to evaporate residual GTA. Cross-linked scaffolds were stored at room temperature in a sealed container until further use.

#### 2.5.3. Scaffolds Physicochemical Characterisation

FTIR: The molecular structure of the synthesised scaffolds was assessed by attenuated total reflectance—Fourier transform infrared (FTIR) spectroscopy. FTIR spectra were recorded over the IR region of 400–4000 cm^−1^ using a Thermo Scientific^TM^ NICOLET^TM^ iS10^TM^ Smart iTX^TM^ FTIR spectrometer (ThermoFisher Scientific, Waltham, MA, USA).

Microarchitecture and porosity: The microarchitecture, pore size, and morphology of the fabricated scaffolds were examined by scanning electron microscopy (SEM). To evaluate the microarchitecture of scaffolds, samples were cross-sectioned and fixed to an adhesive carbon stub. Prior to imaging, samples were sputter coated with an 80:20 gold/palladium mixture to a thickness of around 4 nm using a Cressington 108 auto sputter coater. Imaging was conducted in a Zeiss ULTRA Plus GEMINI^®^ FESEM (Carl Zeiss AG, Jena, Germany) at an accelerating voltage of 2–4 kV.

The porosity of the scaffolds was calculated by measuring the mass of the composite scaffold (m) relative to the density of the individual collagen scaffold (ρ), as described in the following equation:

$$Porosity\;\left(\%\right)=\left(1-\frac{m_{scaffold}}{\rho_{collagen}}\right)\times 100$$

The average diameter of the pores was determined using the captured SEM micrographs. The pore size dimensions were determined as the average of the long and short pore axis of randomly selected pores using the manual mode of the ImageJ analyser. At least 40 pores were assessed for each SEM micrograph.

Mechanical properties: Uniaxial compression testing was conducted to evaluate the effect of nHAp and Mg/nHAp incorporation on the Young’s modulus of the collagen scaffolds. All testing was carried out using a Z050 Zwick Roell mechanical testing machine (Zwick Roell, Ulm, Germany) fitted with 5 N load cell. Samples were pre-hydrated in PBS for 5 min prior to testing and immersed in PBS during testing. Compressive testing was performed at a strain of 10% strain/min, at room temperature. The compressive modulus was calculated within the 2–5% range of the stress–strain curves from four individual samples for each scaffold composition, from three different batches of scaffolds.

Weight loss: Immersion tests were carried out to monitor the degradation behaviour of the scaffolds in PBS. Briefly, samples from each scaffold composition in triplicate were weighed and individually immersed into sterile tubes containing 10 mL of sterile PBS free of Mg^2+^ and Ca^2+^ (21-040-CM, Corning, Corning, NY, USA). The solution had an initial pH of 7.2. Immersed samples were incubated at 37 °C for 1, 3, 5, 7, 14, 21, and 28 days. At each time point, scaffolds were collected and dried at 100 °C for 48 h. The percentage of weight loss of each individual sample was calculated using the following equation:

$$Weight\;loss\;\left(\%\right)=\frac{W_{i}-W_{d}}{W_{i}}\times 100$$

where *W_i_* is the initial weight of the samples before immersion in PBS and *W_d_* is the weight of the samples after degradation in dry state.

Mg^2+^ release: Supernatants resulting from degradation studies were taken to measure the concentration of Mg^2+^ ions released from scaffolds. Supernatants were diluted 1:8 in 5% HNO_3_ and elemental analysis was performed by inductive coupled plasma—optical emission spectroscopy (ICP-OES) using a Spectro Arcos OPI ICP-OES (PerkinElmer, Massachusetts, Hopkinton, MA, USA).

### 2.6. 3D Cell Culture

Prior to in vitro studies, scaffolds were hydrated in a 0.1 M glycine solution for 40 min to block residual aldehyde groups from GTA crosslinking. Scaffolds were sterilised in a 70% EtOH solution for 10 min, followed by two washes in sterile PBS for 10 min. In all steps, 2 mL of solution were used per scaffold. Scaffolds were placed in 24-well suspension plates after hydration and excess liquid was removed. Re-suspended MC3T3-E1 and RAW 264.7 cells (2.5 × 10^5^ cells) were added to the scaffolds, with 10 µL first pipetted onto one side of each scaffold and incubated for 1 h (5% CO_2_, 37 °C) to allow initial cell attachment. Scaffolds were then turned over, and the procedure was repeated on the other side. Each scaffold was seeded with a total of 5 × 10^5^ cells. Fresh media changes were performed every 2–3 days.

#### 2.6.1. Cell Proliferation

Cell proliferation of MC3T3-E1 and RAW 264.7 cells seeded on scaffolds was measured by quantifying DNA content over time using the Quant-iT^TM^ PicoGreen^TM^ dsDNA Assay kit (Invitrogen^TM^). At every desired time point, scaffolds were lysed in 1 mL of 1X assay buffer in dH_2_O containing 1% (*v*/*v*) Triton X-100 and stored at −20 °C. The measurement of cell proliferation was carried out as previously described.

#### 2.6.2. Gene Expression

The gene expression levels of osteoblast markers of osteogenic-induced MC3T3-E1 and RANKL-induced RAW 264.7 cells cultured on scaffolds were analysed by qRT-PCR, following the procedure previously described above. Briefly, scaffolds were washed twice in PBS and lysed by mechanical agitation using a pestle and mortar and 1 mL QIAzol lysis reagent. Extraction of RNA was performed by loading a single silica membrane with a pool of three scaffolds from each individual test sample. For each test sample, either 600 (osteoblasts) or 800 (osteoclasts) ng of purified RNA were reverse transcribed and qRT-PCR analysis was performed as previously described. The mRNA expression levels for each gene of interest were calculated using the ΔΔCt method relative to the reference housekeeping gene GAPDH.

#### 2.6.3. Calcium Quantification

The quantification of cell-mediated calcium deposits was determined using the Calcium (CPC) LiquiColor^®^ Test kit (0150-225, Stanbio, Boerne, TX, USA). Scaffolds were added to 1 mL of 0.5 M HCl lysis buffer and lysed under continuous shaking for 24 h at 4 °C. Samples were assayed in duplicate according to manufacturer’s instructions and absorbance was recorded at 550 nm.

#### 2.6.4. TRAP Activity

The differentiation activity of RANKL-induced RAW 264.7 cells cultured on scaffolds was evaluated by tartrate-resistant acid phosphatase (TRAP) activity. Briefly, 50 μL of cell culture supernatant were mixed with 50 µL of TRAP substrate solution and assayed as previously described. Results are expressed as concentration of TRAP activity (μmol/mL).

## 3. Results

### 3.1. Effect of Mg^2+^ on Pre-Osteoblast and Pre-Osteoclast Cell Viability

**Figure 1:**
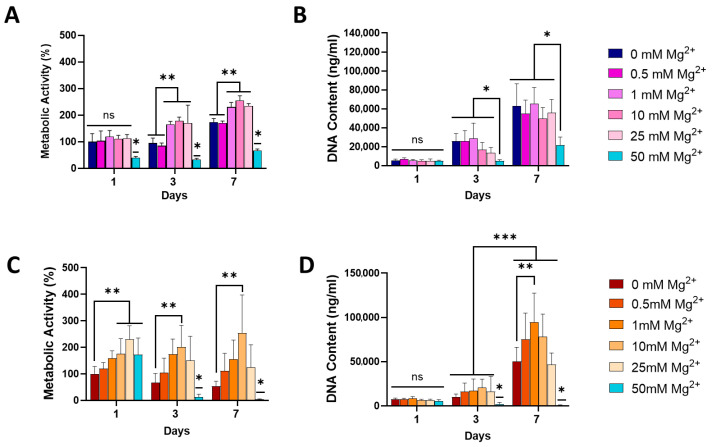
The effect of Mg^2+^ concentration on metabolic activity of MC3T3-E1 (**A**) and RAW 264.7 (**C**) cells and the DNA content of MC3T3-E1 (**B**) and RAW 264.7 (**D**) cells, indicative of proliferation. Error bars represent the standard deviation for *n* = 6. * *p* < 0.05 compared to all groups, ** *p* < 0.05 compared to indicated groups, *** *p* < 0.05 between all groups Day 3 and all groups Day 7, ns *p* > 0.05 (non-significant), as determined by two-way ANOVA.

To determine the effect of Mg^2+^ on osteoblast and osteoclast differentiation, pre-osteoblast MC3T3-E1 and osteoclast progenitor RAW 264.7 cells were cultured with increasing concentrations of extracellular Mg^2+^ (0.5, 1, 10, 25, and 50 mM) and assessed for cell proliferation and metabolic activity at days 1, 3, and 7 post exposure (Figure 1). The metabolic activity of MC3T3-E1 osteoblasts increased over time across all magnesium (Mg^2+^) concentrations, except for the highest concentration of 50 mM (Figure 1A). Notably, 10 and 25 mM Mg^2+^ showed the highest metabolic activity at all time points, indicating that moderate Mg^2+^ concentrations are optimal for promoting osteoblast metabolic function. In contrast, 50 mM Mg^2+^ consistently exhibited the lowest metabolic activity, which was significantly reduced compared to lower Mg^2+^, suggesting potential cytotoxic effects at this high concentration. Similarly, osteoblast proliferation, assessed via DNA content, was significantly decreased after exposure to 50 mM Mg^2+^ by days 3 and 7, while lower concentrations did not have a significant impact (Figure 1B), further suggesting that excessive Mg^2+^ concentrations may impair osteoblast proliferation.

In RAW264.7 osteoclasts, metabolic activity varied across Mg^2+^ concentrations, with 25 mM increasing metabolic activity by days 3 and 7 (Figure 1C). Similarly to what was observed in osteoblasts, 50 mM significantly reduced osteoclast metabolic activity. Interestingly, osteoclast proliferation, as measured by DNA content, was highest at lower Mg^2+^ concentrations (1 mM) by day 7, with no significant increase for higher concentrations (Figure 1D). The lowest proliferation rates were observed at 50 mM Mg^2+^, indicating that elevated Mg^2+^ concentrations may suppress osteoclast proliferation.

Due to the demonstrated cytotoxic effects observed at 50 mM Mg^2+^, for both tested cells, this concentration was not taken forward in further experiments. Therefore, 0.5, 1, 10, and 25 mM were used to study the effect of Mg^2+^ on osteogenesis and osteoclastogenesis.

### 3.2. Effect of Mg^2+^ Increasing Concentration in Osteoblasts Gene Expression, ALP Activity, and Matrix Mineralisation

**Figure 2:**
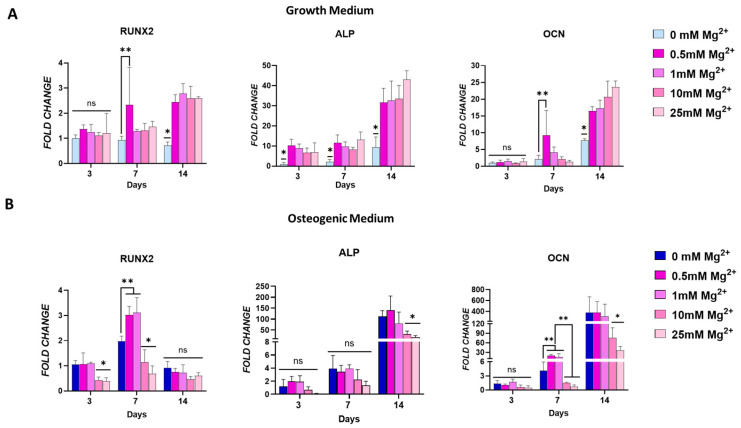

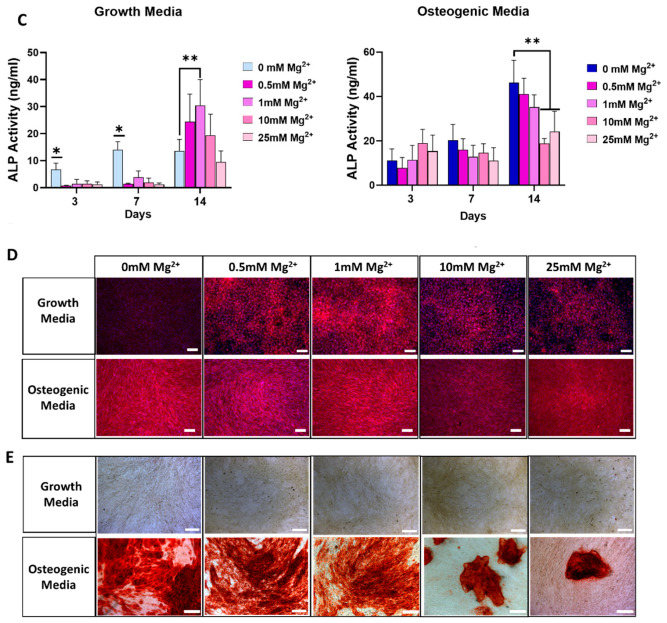
Osteogenic gene expression for RUNX2, alkaline phosphatase (ALP), and osteocalcin (OCN) in osteoblasts cultured in standard growth media (**A**) and osteogenic media (**B**). Effect of Mg^2+^ concentration on ALP activity in both culture conditions (**C**). Images showing ALP staining (**D**) and mineralisation (**E**) as an effect of Mg^2+^ concentration. Error bars represent the standard deviation for *n* = 6. * *p* < 0.05 compared to all groups, ** *p* < 0.05 compared to indicated groups, ns *p* > 0.05 (non-significant), as determined by two-way ANOVA. Scale bars = 150 µm.

To evaluate how an Mg^2+^-enriched microenvironment promotes osteogenic differentiation, the effect of increasing concentrations of extracellular Mg^2+^ was examined on the expression of early- and late-stage osteoblast markers, in both the growth and osteogenic media. In addition, ALP activity and matrix mineralisation were also assessed (Figure 2). In growth media, Mg^2+^ significantly increased the expression of osteoblast markers RUNX2 and ALP over time, with a notable rise observed by day 14. At this time point, significantly higher expression of the transcription factor RUNX2 and downstream genes ALP and OCN was evident in cells exposed to all Mg^2+^ concentrations (Figure 2A). Notably, increasing Mg^2+^ concentrations resulted in a progressive increase in the expression of these markers, highlighting the osteogenic potential of Mg^2+^.

In cells cultured in osteogenic medium, Mg^2+^ enhanced the expression of osteoblast markers at earlier time points (Figure 2B). This enhancement was concentration-dependent, with lower Mg^2+^ concentrations leading to a significant upregulation of RUNX2 by day 7, surpassing the levels achieved with osteogenic factors alone. A similar trend was observed for OCN expression, suggesting that lower Mg^2+^ concentrations may promote an earlier onset of osteogenesis. Interestingly, by day 14, these lower Mg^2+^ concentrations had no impact in RUNX2 and OCN expression, indicating a possible normalisation of early osteogenic signalling over time. In contrast, cells exposed to higher Mg^2+^ concentrations in osteogenic medium showed a marked downregulation of all osteoblast genes’ expression by day 3. This effect was maintained at later time points for ALP and OCN, but not for RUNX2. This suggests that higher Mg^2+^ concentrations may inhibit early osteogenic marker expression in an osteoinductive environment, potentially due to an oversaturation effect.

Mg^2+^ showed an initial inhibitory effect on ALP activity on days 3 and 7 in growth media. This effect was reversed at day 14, when cells exhibited higher ALP activity levels, in a concentration-dependent fashion, peaking at 1 mM and decreasing at higher concentrations. In osteogenic media, exposure to Mg^2+^ did not significantly increase ALP activity at any time point. In fact, higher Mg^2+^ concentrations reduced ALP activity at day 14 (Figure 2C). ALP staining at day 14 further confirmed this trend, where lower Mg^2+^ concentrations in growth media increased ALPs levels, but had no impact in osteogenic media. On the other hand, the effect of higher concentrations in growth media was negligible, while in osteogenic media ALP activity decreased (Figure 2D). Overall, cells exposed to lower Mg^2+^ displayed increased ALP activity, corroborated by ALP staining, compared to cells exposed to higher Mg^2+^ in both media conditions.

To confirm the osteogenic differentiation following exposure to Mg^2+^, cells were evaluated for matrix mineralisation by Alizarin Red staining after 28 days of culture. Interestingly, although exposure to Mg^2+^ in growth media increased the expression of osteogenic markers and ALP activity, this effect did not translate into the final stage of osteogenesis, as shown by the absence of mineral deposits (Figure 2E). In contrast, in cells supplemented with osteogenic factors, large, mineralised areas were visualised. Exposure to lower Mg^2+^ concentrations resulted in similarly sized mineralised areas. However, higher concentrations of Mg^2+^ resulted in a significant reduction in matrix mineralisation, as seen by sporadic mineral deposits after exposure to 10 and 25 mM. This is in line with what was observed regarding osteogenic gene expression and ALP activity in osteogenic media—lower Mg^2+^ concentrations have no impact, while higher Mg^2+^ concentrations significantly reduce it.

### 3.3. Effect of Mg^2+^ Concentration on Osteoclast Markers Expression and Maturation

**Figure 3:**
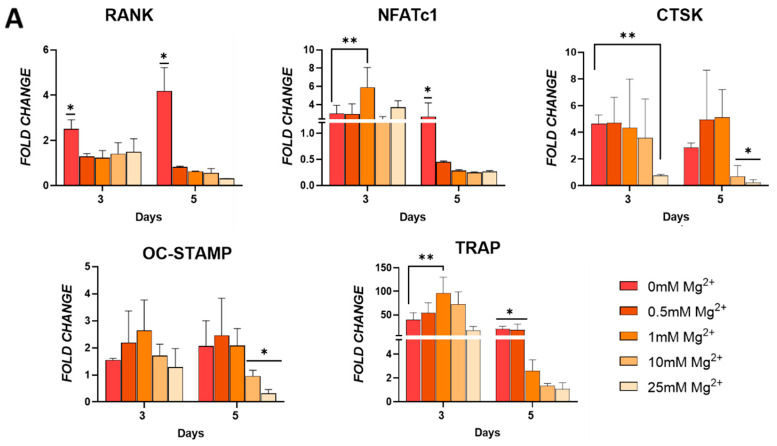

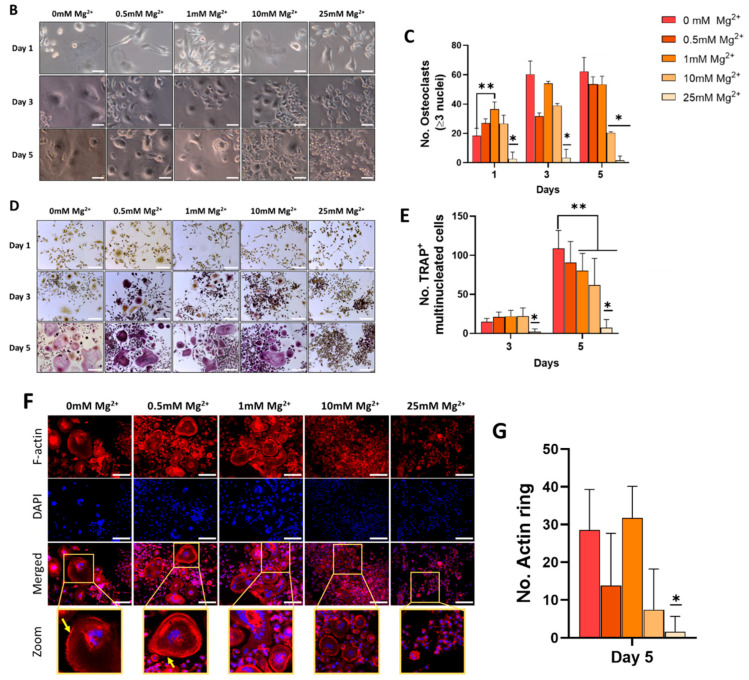
(**A**) Osteoclastic gene expression of RANK, NFATC1, cathepsin K (CTSK), OC-STAMP, and TRAP in cells cultured in osteoclastogenic media. (**B**) Light microscopy and (**C**) quantitative analysis of formation of multinucleated cells indicative of osteoclasts. (**D**) TRAP staining and (**E**) quantification of TRAP positive multinucleated cells. (**F**) F-actin staining of actin rings as indicated by the yellow arrows. (**G**) Quantification of actin rings present. Error bars represent the standard deviation for *n* = 6. * *p* < 0.05 compared to all groups, ** *p* < 0.05 compared to indicated groups, as determined by two-way ANOVA. Scale bars = 150 µm.

To explore the role of Mg^2+^ in regulating osteoclast differentiation, the effect of increasing concentrations on the expression of early- and late-stage osteoclast markers was assessed. Additionally, osteoclast multinucleation and the number of TRAP-positive cells and actin rings was analysed (Figure 3). In the early stages of differentiation (day 3), an increase in the expression of some osteoclast markers was observed. At this time point, cells exposed to a moderate Mg^2+^ concentration, 1 mM, demonstrated elevated expression of osteoclast genes NFATc1 and TRAP, but reduced RANK expression levels (Figure 3A). In fact, expression of RANK was reduced by exposure to any Mg^2+^ concentration. Regarding the expression of OC-STAMP, a marker implicated in the fusion of osteoclast precursors, it was not affected by the presence of Mg^2+^. During osteoclast maturation (day 5), the expression of RANK and NFATc1 significantly decreased in all Mg^2+^ groups. The expression of OC-STAMP, TRAP, and CTSK decreased with increasing Mg^2+^ concentrations and was markedly inhibited in cells exposed to higher Mg^2+^ concentrations.

To determine whether Mg^2+^ could inhibit RANKL-induced osteoclastogenesis, the effect of increasing Mg^2+^ concentrations on osteoclast multinucleation was assessed at days 1, 3, and 5. Cell fusion was visualised in both control cells and those exposed to 0.5 and 1 mM Mg^2+^ from day 1. Representative brightfield images showed a strong inhibitory effect in the fusion of osteoclasts following exposure to 25 and, to a lesser extent, 10 mM Mg^2+^, whereby cells were predominantly mononucleated, particularly at the later time points (Figure 3B). Quantitative analysis demonstrated the formation of multinucleated cells (MNCs) (≥2 nuclei) in all groups, except 25 mM Mg^2+^, at all time points (Figure 3C).

To further evaluate the effect of Mg^2+^ in inhibiting RANKL-induced osteoclastogenesis, the formation of TRAP^+^ cells, a key biochemical marker of osteoclast differentiation and function, was investigated. TRAP staining showed an early formation of TRAP^+^ MNCs in response to concentrations of Mg^2+^ up to 10 mM at day 3, and larger TRAP^+^ MNCs were visualised at day 5. Cells exposed to 25 mM Mg^2+^ repressed the formation of TRAP^+^ MNCs from day 1, indicating an inhibition, from the early stages, of osteoclast differentiation (Figure 3D). Quantitative analysis showed an increase in TRAP^+^ MNCs between days 3 and 5 for all Mg^2+^ concentrations tested, except 25 mM (Figure 3E).

To further determine whether Mg^2+^ inhibited the function of mature osteoclasts, the development of F-actin structures was assessed. Actin cytoskeleton reorganisation was seen in the control group at day 5 (Figure 3F). Exposure to up to 10 mM Mg^2+^ did not significantly change the quantity actin rings, even if they became smaller. However, cells exposed to 25 mM Mg^2+^ were completely devoid of osteoclast morphology, with virtually no actin structures detected (Figure 3G). These results suggest that Mg^2+^ at higher concentrations has the ability to inhibit the function of mature osteoclasts in vitro.

### 3.4. Characterisation of the Composition and Mechanical Properties of Mg^2+^-Loaded Collagen-nHA Scaffolds

Having demonstrated in 2D that 10 and 25 mM Mg^2+^ can successfully inhibit osteoclast differentiation and maturation, but prevent osteogenic differentiation in osteogenic media, we set out to utilise biomimetic Coll/nHA scaffolds as delivery platforms for Mg^2+^, as these scaffolds support bone formation, and the nHA component allows for incorporation and controlled release of Mg^2+^. Compared to simple 2D culture, this scaffold-based delivery may offer a more effective and localised way to enhance the osteogenic effects of Mg^2+^ while maintaining its inhibitory impact on osteoclast activity. We first fabricated collagen-nHA scaffolds containing 10 and 25 mM Mg^2+^ and characterised their composition and mechanical properties.

**Figure 4:**
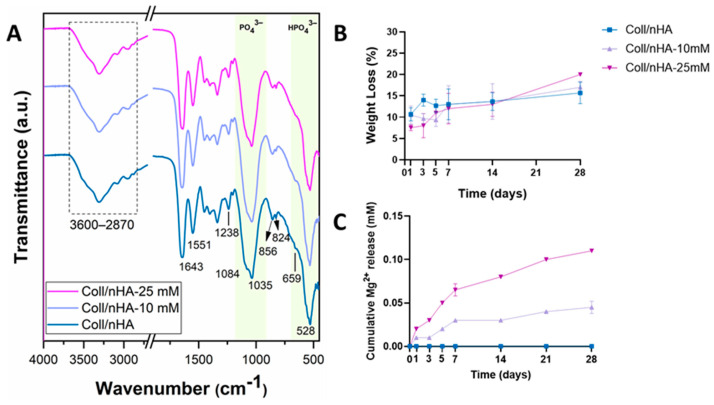
(**A**) ATR-FTIR spectra of freeze-dried scaffolds. (**B**) Weight loss (%) of the scaffolds as a function of time. (**C**) Cumulative Mg^2+^ release as a function of time. Error bars represent the standard deviation for three measurements.

The scaffolds’ composition was determined by FTIR in order to confirm the successful incorporation of Mg^2+^ (Figure 4A). The sharp peaks observed within the fingerprint region (500–1200 cm^−1^) of the Coll/nHA spectra are mainly attributed to the vibrational modes of the PO_4_^3−^ ions. The strong peak at 528 cm^−1^ and shoulder at 659 cm^−1^ are consistent with the interaction between phosphate and hydrogen. The peak at 1035 cm^−1^ corresponds to the asymmetric stretching mode of the P–O bonds. The incorporation of Mg^2+^ caused a minor decrease in the peaks’ intensity, potentially caused by a decrease in apatite crystallinity. Importantly, this did not drastically alter the scaffold structure, as the spectra for both 10 and 25 mM Mg^2+^ resemble that of Coll/nHA. Furthermore, the bands at 2870–3600 cm^−1^, primarily assigned to adsorbed water, were consistent across groups, indicating that bioceramic incorporation did not alter the hydrophilicity of the collagen structure in any of the three scaffold groups.

The percentage weight loss of the scaffolds was evaluated over 28 days under physiological conditions to assess the effect of Mg^2+^ functionalisation on their degradation behaviour (Figure 4B). Increased Mg^2+^ functionalisation resulted in increased weight loss, with the highest percentage weight loss observed in Coll/nHA-25 mM group (Figure 4B). Nonetheless, all scaffold groups exhibited a slow degradation profile with approximately 80% remaining after 28 days of immersion, indicating the incorporation of Mg did not significantly affect degradation.

To assess the delivery capability of the developed Coll/nHA-Mg scaffolds, cumulative release of Mg^2+^ was measured over 28 days in PBS under physiological conditions. Both Coll/nHA-10 mM and Coll/nHA-25 mM scaffolds displayed a similar release pattern, with a burst release until day 7, followed by a sustained increase from that point forward. No Mg was released from the Coll/nHA scaffolds. At each time point, the amount of Mg^2+^ released from the Coll/nHA-25 mM scaffolds was consistently higher than that from the Coll/nHA-10 mM, further confirming the successful incorporation of Mg within the scaffold structure. Interestingly, for both formulations, the released concentration is only a small fraction (<1%) of the total incorporated Mg^2+^.

**Figure 5:**
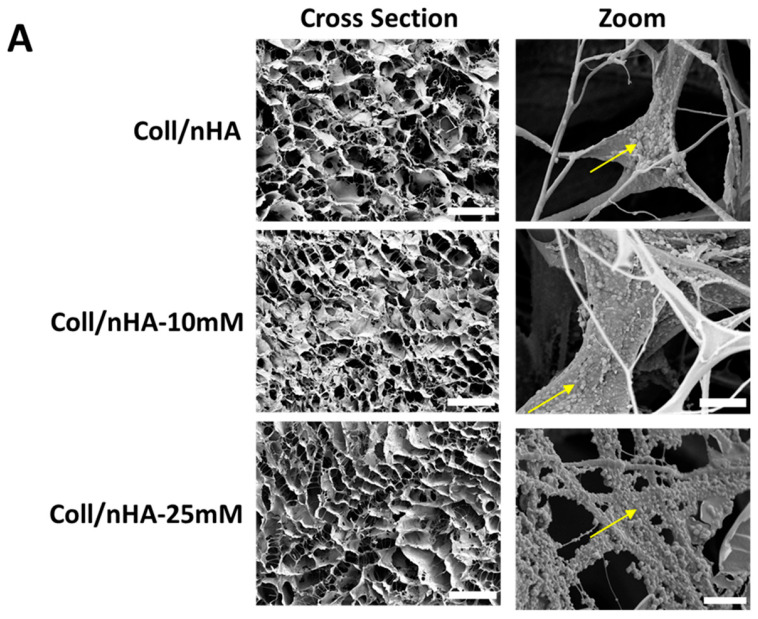

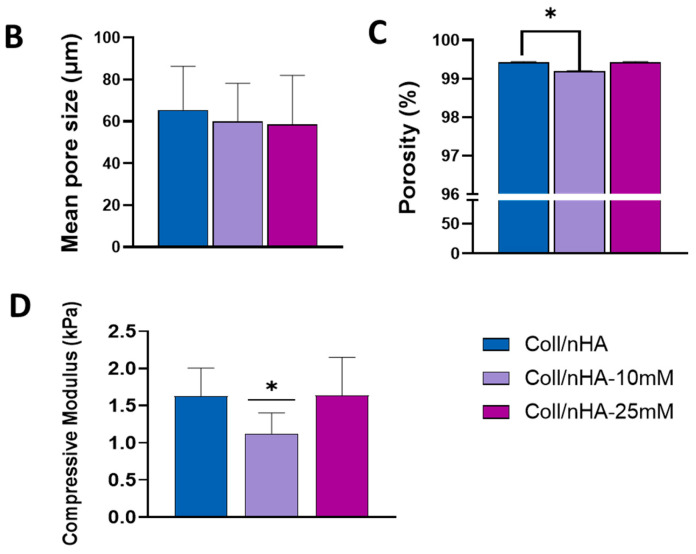
(**A**) Representative SEM micrograph of Coll/nHA scaffolds demonstrating the internal pore structure and the incorporation of nHA within the collagen matrix (yellow arrows). Scale bar = 200 µm in cross-section. Scale bar = 50 µm in zoom. The effect of Mg^2+^ functionalisation of nHA on (**B**) mean pore size, (**C**) porosity, and (**D**) compressive modulus of the range of scaffolds. The error bars represent the standard deviation for three measurements. * *p* < 0.05 compared to all groups, as determined by unpaired, two-tailed *t*-test.

To further assess the impact of Mg^2+^ incorporation on scaffolds, their mechanical properties and microarchitectures were assessed (Figure 5). The effect of Mg^2+^ functionalisation on scaffold porosity and microarchitecture was evaluated by SEM analysis. SEM micrographs revealed that all scaffolds, independently of Mg^2+^ content, exhibited a highly porous and interconnected network, with nHA particles aligned alongside the collagen fibres (Figure 5A). Quantitative analysis confirmed that Mg^2+^ functionalisation did not significantly affect the mean pore size of the scaffolds, which remained at ~60 µm (Figure 5B). Similarly, the Mg^2+^ functionalisation did not affect the porosity of the scaffolds. All scaffold groups remained highly porous, with values of above 98% (Figure 5C). A lower compressive modulus was observed in the Coll/nHA-10 mM scaffolds compared to the Coll/nHA and Coll/nHA-25 mM groups; however, this decrease in mechanical properties was non-significant (Figure 5D).

### 3.5. Effect of Mg^2+^-Containing Scaffolds on Pre-Osteoblast and Pre-Osteoclast Cell Viability and Differentiation

**Figure 6:**
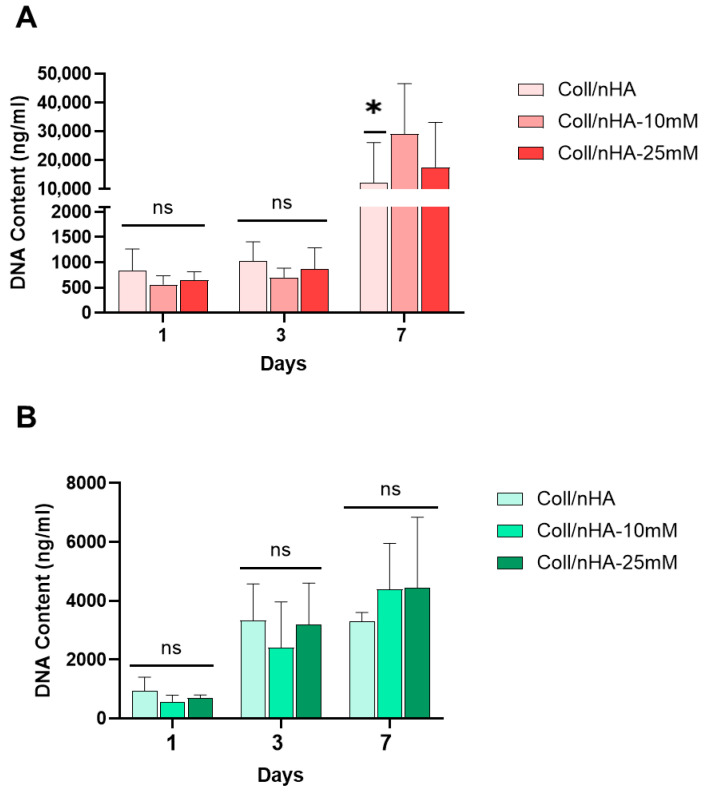
The effect of Mg^2+^ on MC3T3-E1 (**A**) and RAW 264.7 (**B**) cell proliferation within Coll/nHA scaffolds. Error bars represent the standard deviation for *n* = 6. * *p* < 0.05 compared to all groups, ns *p* > 0.05 (non-significant), as determined by two-way ANOVA.

The effect of Mg^2+^ on pre-osteoblast (MC3T3-E1) and pre-osteoclast (RAW 264.7) cell proliferation within the scaffolds was assessed (Figure 6). No significant difference in MC3T3-E1 DNA content was observed between the scaffold groups at days 1 and 3. However, by day 7, a significant increase in DNA content was observed in the Coll/nHA-10 mM and Coll/nHA-25 mM Mg^2+^ scaffold groups compared to the Mg^2+^-free Coll/nHA group, indicating an increase in MC3T3-E1 proliferation as an effect of Mg^2+^ functionalisation (Figure 6A). RAW 264.7 cell proliferation was observed across the 7-day culture period, with increasing DNA content quantified in all scaffold groups. However, no significant differences in proliferation were observed between scaffold groups (Figure 6B).

**Figure 7:**
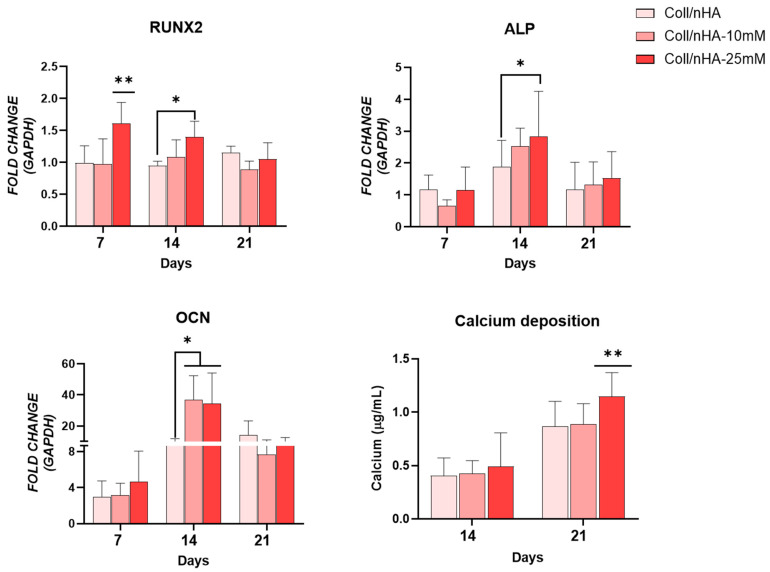
Osteogenic gene expression for RUNX2, alkaline phosphatase (ALP) and osteocalcin (OCN), and calcium mineralization in scaffolds up to day 21. Error bars represent the standard deviation for *n* = 6. * *p* < 0.05 compared to control group, ** *p* < 0.05 compared to all groups, as determined by two-way ANOVA.

Next, we assessed the potential of Mg^2+^-functionalised scaffolds to promote osteoblast differentiation and evaluated whether delivery of Mg^2+^ via Coll/nHA scaffolds could negate the inhibitory effect on osteogenesis observed in 2D culture (Figure 7). An early increased expression in RUNX2 was observed at day 7 in the Coll/nHA-25 mM Mg^2+^ scaffold group compared to the other scaffold groups. By day 14 this increase was only significant compared to the Mg^2+^-free Coll/nHA group. ALP expression peaked at day 14 in all scaffold groups. However, the Coll/nHA-25 mM Mg^2+^ scaffolds demonstrated a significant increase in ALP expression at this time point compared to the Mg^2+^-free Coll/nHA scaffolds. There was no significant difference in OCN expression between the Coll/nHA-10 mM and Coll/nHA-25 mM scaffolds. Nevertheless, OCN gene expression was significantly upregulated at day 14 in both 10 mM and 25 mM Mg^2+^ scaffolds compared to Mg^2+^-free Coll/nHA group. An increase in calcium deposition was observed in all scaffold groups between days 14 and 21. At day 21, the Coll/nHA-25 mM had a significant increase in calcium deposition compared to the other scaffold groups.

**Figure 8:**
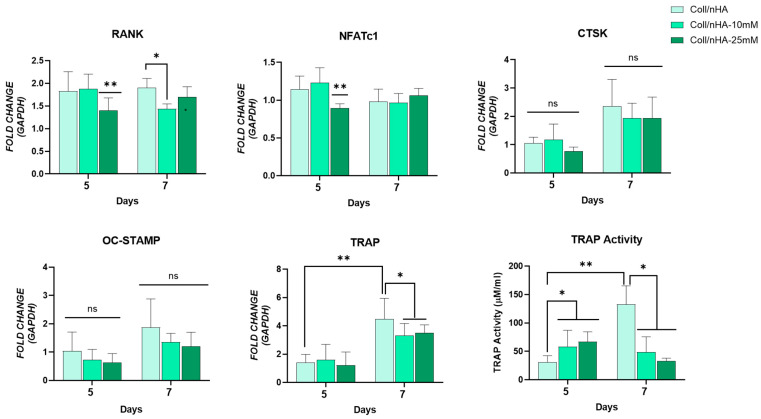
Osteoclastic gene expression RANK, NFATc1, cathepsin K (CTSK), OC-STAMP, TRAP, and quantification of TRAP activity. Error bars represent the standard deviation for *n* = 6. * *p* < 0.05 compared to control group, ** *p* < 0.05 compared to all other scaffold groups, ns *p* > 0.05 (non-significant) as determined by two-way ANOVA.

The potential of the Mg^2+^-functionalised Coll/nHA scaffolds to inhibit osteoclastogenesis was also assessed (Figure 8). Mg^2+^-functionalised Coll/nHA scaffolds reduced RANK, NFATc1, and TRAP gene expression. Interestingly, no significant difference in CTSK and OC-STAMP gene expression was observed between any of the scaffold groups. An early and significant reduction in RANK was observed at day 5 in the Coll/nHA-25 mM scaffolds compared to the other scaffold groups. By day 7, the Coll/nHA-10 mM scaffolds demonstrated significantly reduced expression compared to the Mg^2+^-free Coll/nHA scaffold group. Similarly, the Coll/nHA-25 mM scaffolds significantly reduced NFATc1 expression at day 5 compared to the other scaffold groups. However, by day 7, no significant difference was observed. A significant increase in TRAP expression between days 5 and 7 was observed in the Mg^2+^-free Coll/nHA scaffolds. This effect was inhibited in the 10 and 25 mM Coll/nHA groups, with both scaffold groups demonstrating significantly decreased TRAP expression at day 7 compared to the Mg^2+^-free Coll/nHA group. TRAP activity was assessed as a measure of osteoclast maturation and function. Similarly to TRAP gene expression, a significant increase in TRAP activity between days 5 and 7 was observed in the Mg^2+^-free Coll/nHA scaffolds. While 10- and 25-mM scaffold groups demonstrated a small but significant increase in TRAP activity at day 5 compared to the Mg^2+^-free Coll/nHA scaffolds, by day 7 the Mg^2+^-functionalised Coll/nHA scaffolds demonstrated a significant reduction in TRAP activity compared to the Mg^2+^-free Coll/nHA scaffolds, indicating their potential to inhibit osteoclast differentiation.

## 4. Discussion

High local magnesium concentration has been shown to both promote bone formation and growth and reduce osteoclast maturation and activity; thus, bone resorption results in accelerated bone regeneration. However, achieving these effects simultaneously remains elusive, as it is not clear whether a “one size fits all” concentration exists. In this study, the effects of varying extracellular Mg^2+^ concentrations on the differentiation and extracellular matrix mineralisation of pre-osteoblasts, as well as in the inhibition of osteoclastogenesis in monocytes were assessed. Firstly, the therapeutic window of Mg^2+^ was determined by assessing metabolic activity and cell proliferation over a 7-day period. Only the highest Mg^2+^ concentration tested, 50 mM, resulted in a significant reduction in both proliferation and metabolic activity. In a study published by Zhang et al., concentrations as high as 20 mM were shown to be biocompatible in RAW 264.7 cells [42]. However, for pre-osteoblasts, MSCs, and endothelial cells, Mg^2+^ concentrations around 20 mM have been reported to reduce cell viability [43,44,45]. While these differences can be attributed to cell type sensibility, they highlight the existence of conflicting reports in the literature regarding Mg^2+^ cytotoxic limitations, and the importance of addressing this issue by establishing a therapeutic biocompatible window for Mg^2+^.

In order to thoroughly assess the impact of Mg^2+^ in osteogenesis, the commitment of progenitor cells into osteoblasts through osteogenic differentiation needs to be assessed at different levels. Firstly, the upregulation of early- and late-stage osteogenic marker expression can indicate a commitment of progenitor cells into the osteogenic lineage. RUNX2 is a well-established master regulator of osteogenic differentiation, making its early expression essential for the commitment of progenitor cells to the osteogenic phenotype and for the downstream signalling of osteogenic markers. Our data point to Mg^2+^ increasing RUNX2 expression in growth media, but only after 14 days, contrary to what has been reported, for both MC3T3-E1 [46] and mesenchymal stem cells [47], where increased RUNX2 expression was observed after 7 days. Expression of downstream osteogenic genes ALP and OCN was increased at day 14 in growth media after exposure to every Mg^2+^ concentration, pointing to an Mg^2+^-dependent osteogenic differentiation. Interestingly, when cultured in osteogenic media, low concentrations (0.5 and 1 mM) of Mg^2+^ further promoted RUNX2 expression at day 7 but reduced it at day 14, in line with what has been reported previously [48]. However, expression of downstream genes ALP and OCN in osteogenic media was not increased by exposure to Mg^2+^. In fact, a trend was observed whereby increasing Mg^2+^ concentration decreased the expression of osteogenic markers compared to the positive control. This points to the capability of Mg^2+^ to drive osteogenesis in the absence of osteogenic factors but to have an inhibitory effect on these cells in their presence. This is the opposite of what has been reported, as a synergistic effect between differentiation media and extracellular Mg^2+^ was observed in mesenchymal stem cells regarding these markers’ expression [47]. Moreover, it reinforces the concentration- and time-dependent fashion in which magnesium affects osteogenesis.

An increase in osteogenic gene expression does not necessarily correlate with a full commitment to osteogenic differentiation. It is important to assess the further downstream expression of proteins known to be pivotal in the osteogenesis differentiation process. One such protein is ALP. In this study, we show that Mg^2+^ increases ALP activity at day 14, peaking at 1 mM, compared to growth media. However, in the presence of osteogenic factors, increases in Mg^2+^ concentrations resulted in a decrease in ALP activity. Interestingly, elsewhere it has been reported that exposure to Mg^2+^ decreases ALP activity, but when in conjunction with osteogenic factors, there was a synergistic effect observed [46].

Finally, it is important to assess whether the overexpression of osteogenic factors and proteins leads to matrix mineralisation, as it is the definite display of successful osteogenic differentiation. Despite the upregulated expression of osteogenic genes in cells within growth media, there were no signs of matrix mineralisation following exposure to any Mg^2+^ concentration. Conversely, in osteogenic media, 0.5 and 1 mM Mg^2+^ were able to achieve similar mineralisation as differentiation media alone. Yet, osteogenic-induced cells exposed to higher Mg^2+^ exhibited a significant reduction in matrix mineralisation. These findings indicate that an Mg^2+^ enriched environment can stimulate osteogenic differentiation in a time- and concentration-dependent manner and demonstrate that an Mg^2+^ concentration between 0.5 and 1 mM is optimal for constructing an osteogenic microenvironment. This is partially in line with the literature, as it has been shown that concentrations around 5 mM of Mg^2+^ will result in inhibited matrix mineralisation [49]. However, contradictory results have been reported. For instance, the very same concentration, in osteogenic media, has been shown to both increase mineralisation [48] and to almost completely inhibit it [50]. This lack of consensus highlights the importance of determining the optimal range of Mg^2+^ concentration for osteogenesis promotion in specific experimental setups.

The apparent contradiction between osteogenic gene expression and matrix mineralisation, particularly in the absence of osteogenic factors, can be explained by the limited osteogenic potential of the pre-osteoblastic MC3T3-E1 cell line in such conditions, particularly when compared with other precursors, such as MSCs [51]. Nevertheless, the fact that low concentrations of Mg^2+^ in osteogenic media were able to replicate the levels of matrix mineralisation points to the ion’s incapability to, by itself, promote osteogenesis. It is important to note the lack of literature regarding the effects of Mg^2+^ on osteogenesis in the presence of osteogenic factors, as studies mostly focus on the use of this metal as a replacement for such factors rather than an adjuvant. Moreover, the majority of current research is carried out with other osteogenic precursors, namely MSCs, likely due to their heightened osteogenic potential.

This study also demonstrated that Mg^2+^-driven inhibition of osteoclast gene expression was dependent on both time and concentration. For instance, in the early stages of differentiation, an increase in the expression of osteoclast markers was observed. Interestingly, expression of transcription factor NFATc1 followed a similar trend to RANK, with no decrease in expression being observed even at the highest Mg^2+^ concentrations. This indicates that osteoclast differentiation progresses downstream of RANK signalling independently of Mg^2+^ stimulation. However, during osteoclast maturation, expression of these factors was heavily downregulated with exposure to higher Mg^2+^ concentrations (10 and 25 mM). Importantly, this translated into a reduction in late markers OC-STAMP, TRAP, and CTSK expression, showing the potential of Mg^2+^ to inhibit osteoclastogenesis. This is in line with the literature, as it has been shown that increasing Mg^2+^ concentrations reduce the expression of these osteoclast markers [52]. Interestingly, it has also been reported that Mg^2+^ deficiency in cell media augmented osteoclastogenesis, regarding both increased osteoclast markers expression and higher number of osteoclast-like cells [53]. Previous studies found that increasing concentrations of a Mg extract, up to 6 mM, favoured osteoclast differentiation, while higher concentrations inhibited it [52].

In order to confirm that the reduction in osteoclastogenic genes resulted in the inhibition of osteoclast differentiation and maturation, we assessed the formation of multinucleated cells expressing TRAP, a key structural feature of osteoclastogenesis. It has been shown that only the highest Mg^2+^ concentration tested, 25 mM, reduced the number of TRAP^+^ cells and TRAP activity consistently over time. Previously reported results showed that Mg^2+^, at low concentrations, can in fact lead to a higher number of osteoclasts and TRAP activity, while higher concentrations led to a similar decrease in both the number of osteoclasts and TRAP activity [54]. In similar fashion, other mature osteoclast features—number of actin rings, nuclei per osteoclast, and the cell size—all reduced when exposed to 10 mM and, in particular, 25 mM Mg^2+^ [55]. These results strengthen the hypothesis that high Mg^2+^ concentrations are needed for successful osteoclastogenesis inhibition and demonstrate the effect of Mg^2+^ in modulating osteoblast and osteoclast differentiation and activity at both the gene and protein level. Importantly, a single concentration that simultaneously promoted osteogenesis and inhibited osteoclastogenesis was not found. Moreover, while osteogenic genes had their expression upregulated by lower Mg^2+^ concentrations in growth media, this did not translate into matrix mineralization. Conversely, the ideal concentrations to inhibit osteoclastogenesis (from 10 to 25 mM) are not suitable to promote osteogenesis, as they reduce the expression of osteogenic markers.

An avenue that can potentially be used to address this issue is through the use of local delivery systems. Herein, we propose that a sustained release mechanism could simultaneously promote osteogenesis and inhibit osteoclastogenesis. Collagen-based scaffolds have been extensively used and characterised in the context of bone regeneration due to the protein’s role in biological bone tissue, as well as its ability to provide a suitable environment for osteogenesis and bone formation [56,57,58,59]. Nanohydroxyapatite (nHA) is well-established as an osteogenic material. Moreover, its structure allows for simple and straightforward ionic substitution, allowing the incorporation of ions such as magnesium [60,61]. By functionalising the nHA phase of Coll/nHA with Mg^2+^, we hypothesised that osteogenesis could be enhanced while still maintaining the anti-osteoclastogenic effect of Mg^2+^ observed in 2D.

Coll/nHA scaffolds were successfully functionalised with 10 and 25 mM Mg^2+^, as suggested by a decrease in the FTIR absorption band at 1035 cm^−1^ corresponding to P–O stretching, indicating possible interaction between Mg^2+^ and the phosphate groups of nHA. In general, the degradation of all scaffold groups remained slow and controlled, a necessary feature to support sustained tissue regeneration [62]. Nevertheless, the incorporation of Mg within the scaffolds resulted in a slightly delayed degradation, similar to what has been reported elsewhere [63,64]. This slower degradation is highly desirable, as it allows the cells to be exposed to a constant Mg^2+^ concentration. This improves cellular response regarding motility and migration and avoids the impairment of osteoblast activity that comes from high Mg^2+^ concentration [65]. Notably, Mg^2+^ incorporation, independently of concentration, did not significantly alter the porosity, pore size, or compressive modulus of the scaffolds, ensuring that any observed in-cell activity is due to the biological impact of Mg^2+^ and nHA, not the mechanical and structural properties of the scaffolds.

Proliferation of pre-osteoblasts was not impacted by the presence of Mg^2+^ at earlier time points and was, in fact, increased by day 7. On the other hand, the DNA content of pre-osteoclast cells was the same at all time points for all scaffolds tested. This not only validates the therapeutic window defined in 2D experiments, as it also is in accordance with previously reported results [42,64].

Interestingly, only 25, but not 10 mM Mg^2+^, scaffolds resulted in an increased expression of osteogenic genes. In particular, RUNX2, the master regulator and early-stage marker of osteogenesis, was overexpressed by day 7, while in 2D experiments such overexpression was only detected at day 14. Late-stage markers ALP and OCN genes were overexpressed at day 14, pointing to a successful commitment to osteogenic differentiation. Consistent with this, Minardi et al. [66] have shown that magnesium plays a crucial role in the promotion of osteogenesis. A significant increase in the expression of osteogenesis-associated markers ALP and OCN was found in cells grown in Coll/nHA scaffolds functionalised with Mg compared with the levels observed in Coll/nHA scaffolds without magnesium. Interestingly, OCN expression was higher than even in optimised and well-established 2D protocols. Importantly, and contrary to what was observed in 2D Mg^2+^ exposure, matrix mineralisation was observed when using the Coll/nHA scaffold as the ion delivery system, as calcium deposition was significantly increased by day 21 compared to control scaffolds. Calabrese et al. [67] have shown that MSCs cultured in these scaffolds deposit high quantities of calcium over time when in the presence of osteogenic factors. However, the scaffolds alone in non-inductive media are per se sufficient to stimulate osteogenic differentiation. This highlights not only the well-established osteogenic nature of Coll/nHA scaffolds [38], but also the improvement that can be achieved by the adequate concentration and dosage regimen of osteogenic factors such as Mg^2+^.

The impact of Mg^2+^-loaded scaffolds on osteoclastogenic gene expression was varied. CTSK and OC-STAMP expression was not altered. Both 10 and 25 mM Mg^2+^ reduced RANK expression, while NFATc1 levels only decreased with 25 mM Mg^2+^ scaffolds. TRAP gene expression increased in all scaffold groups from day 5 to day 7. However, this increase was significantly lower in 10 and 25 Mg^2+^ than in magnesium-free scaffolds. Following a similar pattern, TRAP activity at day 5 was higher in the presence of magnesium but did not increase further at day 7. On the other hand, Coll/nHA scaffolds were not capable of inhibiting the increase in TRAP activity over time, resulting in a significantly higher level when compared to both Mg^2+^ concentrations, highlighting the vital role that magnesium plays in the inhibition of osteoclastogenesis. The majority of research regarding the use of Coll/nHA-based scaffolds for bone tissue engineering focuses on their osteogenic potential, and their direct impact on osteoclastogenesis has been scarcely studied. Nevertheless, it has been reported that Coll/nHA scaffolds alone can indirectly decrease osteoclastogenesis by promoting osteoblast activation and reducing the number of inflammatory and osteoclast precursor cells [68,69]. Similarly, scaffolds containing magnesium have been shown to reduce bone resorption and inhibit osteoclastogenesis via immunomodulation, namely the suppression of the RANK pathway in macrophages [70]. In addition, Muller et al. [71] have shown that the increase in local pH due to Mg^2+^ sustained release also suppresses osteoclastogenesis. Our data point to similar findings, as the incorporation, and sustained release of Mg^2+^ in the scaffolds resulted in a reduction of key osteoclastogenic markers. Notably, unlike the 2D results, one single concentration of Mg^2+^ incorporated in the scaffolds was able to simultaneously promote osteogenesis and inhibit osteoclastogenesis.

Importantly, the amount of Mg^2+^ released when loaded on collagen-based scaffolds via nHA was lower than what the cells experienced in 2D culture, which interestingly led to an enhanced osteogenesis. While the use of nHA as a delivery vehicle may have contributed to such an increase in mineralisation, as nHA is itself osteogenic, the presence of Mg^2+^ further enhanced osteogenic differentiation. Moreover, osteoclast maturation and function were successfully inhibited. The optimal range of concentrations varies as a function of the experimental setup (cell types, time points analysed, etc.). In fact, even the exposure approach for Mg^2+^, such as different magnesium alloys, can have a strong impact on the osteogenic/osteoclastogenic outcomes [72]. The materials herein produced and characterised promoted cellular proliferation of osteoblasts, but not osteoclasts. Importantly, the slow-release profile of Mg^2+^ 25 mM scaffolds simultaneously promoted osteogenesis and inhibited the expression of key osteoclastogenesis genes compared to Coll/nHA-only scaffolds. While the osteogenic impact of nHA is indisputable, particularly for osteogenesis promotion, the small concentration of Mg^2+^ released further enhanced this effect. The improved understanding of the concentrated Mg^2+^ microenvironment in modulating bone cell behaviour herein discussed may enlighten the design of orthopaedic implants with osteoclastogenic-modulatory abilities for bone regeneration.

## 5. Conclusions

The data presented is this study provide insight into the spatiotemporal effect of increasing concentrations of Mg^2+^ in modulating osteoblast and osteoclast differentiation and activity. The results show that, in 2D, no single concentration of Mg^2+^ is capable of promoting osteogenesis and inhibiting osteoclastogenesis in vitro. However, when loaded on collagen-based scaffolds via nHA, the amount released was lower than what the cells experienced in 2D culture, resulting in enhanced osteogenesis, while simultaneously inhibiting osteoclastogenesis, highlighting the need for scaffold-based strategies to modulate the local ionic environment. Importantly, factors such as cell types, time points analysed, or genetic markers assessed need to be taken into account when analysing data and comparing it with the literature. For instance, most of the literature regarding the impact of Mg^2+^ in osteogenics is performed in mesenchymal cells and not MC3T3-E1. In this light, further studies, such as incorporating co-culture experiments, would allow a better understanding of the impact that osteoblast-secreted factors, namely RANKL, have on osteoclastogenesis. Similarly, the impact of Mg^2+^ in a 3D environment could be further evaluated by measuring local concentration gradients at the cell–material interface, providing insight into release kinetics and informing in vivo studies to realise the translational potential of these scaffolds. Nevertheless, the findings presented provide a new standpoint for optimised Mg^2+^ concentration and dosing regimen for inducing osteogenesis and impeding osteoclastogenesis.

## Figures and Tables

**Table 1:** Stoichiometric ratio of Mg substituted nHA.

Sample	MgCl_2_ (M)	Ca(NO_3_)_2_·4H_2_O (M)	(NH_4_)_2_)HPO_4_ (M)	Mg/(Mg + Ca) mol (%)	(Mg + Ca)/P Ratio
**nHA**	–	1	0.6	0	1.67
**10 Mg/nHA**	0.1	0.9	0.6	10	1.67
**25 Mg/nHA**	0.25	0.75	0.6	25	1.67

## Data Availability

The original contributions presented in the study are included in the article, further inquiries can be directed to the corresponding author.
